# Clinical significance and different strategies for re-elevation of plasma EBV-DNA during treatment in pediatric EBV-associated hemophagocytic lymphohistiocytosis

**DOI:** 10.1016/j.jped.2024.03.006

**Published:** 2024-04-08

**Authors:** Wenzhi Zhang, Yun Peng, Yining Qiu, Li Cheng, Yuhong Yin, Ying Li, Lizhen Zhao, Xiaoyan Wu

**Affiliations:** Huazhong University of Science and Technology, Tongji Medical College, Union Hospital, Department of Pediatrics, Wuhan, China

**Keywords:** Hemophagocytic lymphohistiocytosis, EBV infection, EBV-DNA, Treatment, Prognosis

## Abstract

**Objective:**

Monitoring the disease status of Epstein–Barr virus (EBV)–related hemophagocytic lymphohistiocytosis (HLH) patients is crucial. This study aimed to investigate the different strategies and outcomes of patients with EBV-HLH and re-elevated EBV-DNA.

**Method:**

A retrospective analysis was conducted on 20 patients diagnosed with EBV-HLH. Clinical features, laboratory tests, treatments, plasma EBV-DNA levels, and outcomes were assessed. Three cases were highlighted for detailed analysis.

**Results:**

Nine of the 20 patients had a re-elevation of EBV-DNA during treatment, and 55.5 % (5/9) experienced relapses. Patients with persistently positive plasma EBV-DNA (*n* = 4) and those with re-elevated EBV-DNA after conversion (*n* = 9) showed a significantly higher relapse rate compared to those with persistently negative EBV-HLH (*n* = 7) (*p* < 0.05). Among the highlighted cases, Case 1 exhibited plasma EBV-DNA re-elevation after four weeks of treatment without relapse, maintaining stability with the original treatment regimen, and eventually, his plasma EBV-DNA turned negative. In Case 2, plasma EBV-DNA was elevated again with a recurrence of HLH after L-DEP. Consequently, she underwent allogeneic hematopoietic stem cell transplantation and eventually achieved complete remission (CR) with negative plasma EBV-DNA. Case 3 experienced plasma EBV-DNA re-elevation after L-DEP but remained in CR, discontinuing chemotherapy without relapse.

**Conclusion:**

The re-elevation of plasma EBV-DNA during EBV-HLH treatment poses challenges in determining disease status and treatment strategies. Optimal management decisions require a combination of the level of elevated EBV-DNA, the intensity of hyperinflammation, and the patient's immune function.

## Introduction

Hemophagocytic lymphohistiocytosis (HLH) is a group of hyperinflammatory syndromes caused by primary or secondary immune abnormalities, characterized by persistent fever, hepatosplenomegaly, hemocytopenia, and hypofibrinogenemia. Epstein–Barr virus (EBV) infection is the most common trigger for secondary HLH worldwide, especially in Asian countries, including China.^1^^,^^2^ Without appropriate therapy, patients with EBV-HLH have a high mortality rate.

EBV is the most common secondary factor of HLH, as well as a trigger of family HLH, and is closely related to NK/T lymphoma HLH. The detection of EBV-DNA is crucial in the evaluation of EBV-associated diseases, particularly EBV-HLH. However, current research on treatment choices following a re-elevation in EBV-DNA levels remains limited.^3^, ^4^, ^5^ In addition to controlling the inflammatory storm, EBV elimination is also an essential therapeutic goal for EBV-HLH patients.^6^ Several studies have demonstrated that higher EBV-DNA load before treatment and EBV-DNA load of more than 10^3^ copies/mL after treatment are associated with poor prognosis.^5^^,^^7^^,^^8^ However, previous studies have not clarified whether the re-elevation of EBV-DNA during treatment indicates progression and the need for intensive treatment. To investigate the relationship between the re-elevation of plasma EBV-DNA and disease status, and to provide a basis for choosing an appropriate treatment for EBV-HLH patients, the authors analyzed the clinical data of 20 cases of EBV-HLH. Periodic monitoring of EBV-DNA in plasma and peripheral blood mononuclear cells (PBMC) was performed in EBV-HLH patients. In this study, the authors highlighted the different management strategies and outcomes observed in three EBV-HLH patients who experienced a re-elevation of plasma EBV-DNA during treatment.

## Methods

### Patients

This study was approved by the Ethics Committee of the Union Hospital of Tongji Medical College, Huazhong University of Science and Technology (NO.2023-0253). This retrospective study enrolled 20 patients with EBV-HLH admitted to Wuhan Union Hospital between January 2018 and July 2022, and three of these patients were highlighted. All patients met the HLH-2004 diagnostic criteria as well as the diagnostic criteria for EBV infection.^9^^,^^10^ Patients who did not receive chemotherapy after diagnosis were excluded. Clinical and laboratory data, treatment, and outcomes of the patients were analyzed.

### Treatment and evaluation

According to therapy guideline for HLH, the therapy strategy for patients with EBV-HLH included etoposide-based protocol which based on HLH-1994 and HLH-2004 protocol (etoposide 100 mg/m^2^/dose, once/week. methylprednisolone 10 mg/kg/d * 3 days, 5 mg/kg/d * 3 days, 2 mg/kg/d * 8 days, followed by a gradual 8-week taper), and those who did not achieve complete response (CR) at 4 weeks were treated with the DEP/L-DEP regimen, which included liposomal injection of adriamycin hydrochloride (25 mg/m^2^, day 1), etoposide (100 mg/m^2^ once weekly on days 1, 8, and 15), and methylprednisolone (15 mg/kg on days 1–3; 2 mg/kg on days 4–6; tapered off for 3 weeks).

Treatment response evaluations were performed at 2, 4, and 8 weeks of therapy. Response to treatment has been defined as follows: complete response (CR) has been defined as a complete resolution of fever, serum ferritin levels, triglyceride (TG), fibrinogen (FIB), Soluble interleukin-2 receptor alpha chain (sCD25), and blood cell count return to normal; and partial response (PR) has been defined as an improvement of at least 25 % in ≥ 2 symptoms/laboratory indicators. Relapse has been defined as the recurrence of fever associated with both increased serum ferritin levels and EBV-DNA copies in peripheral blood.

### EBV-DNA detection

**Figure 1:**
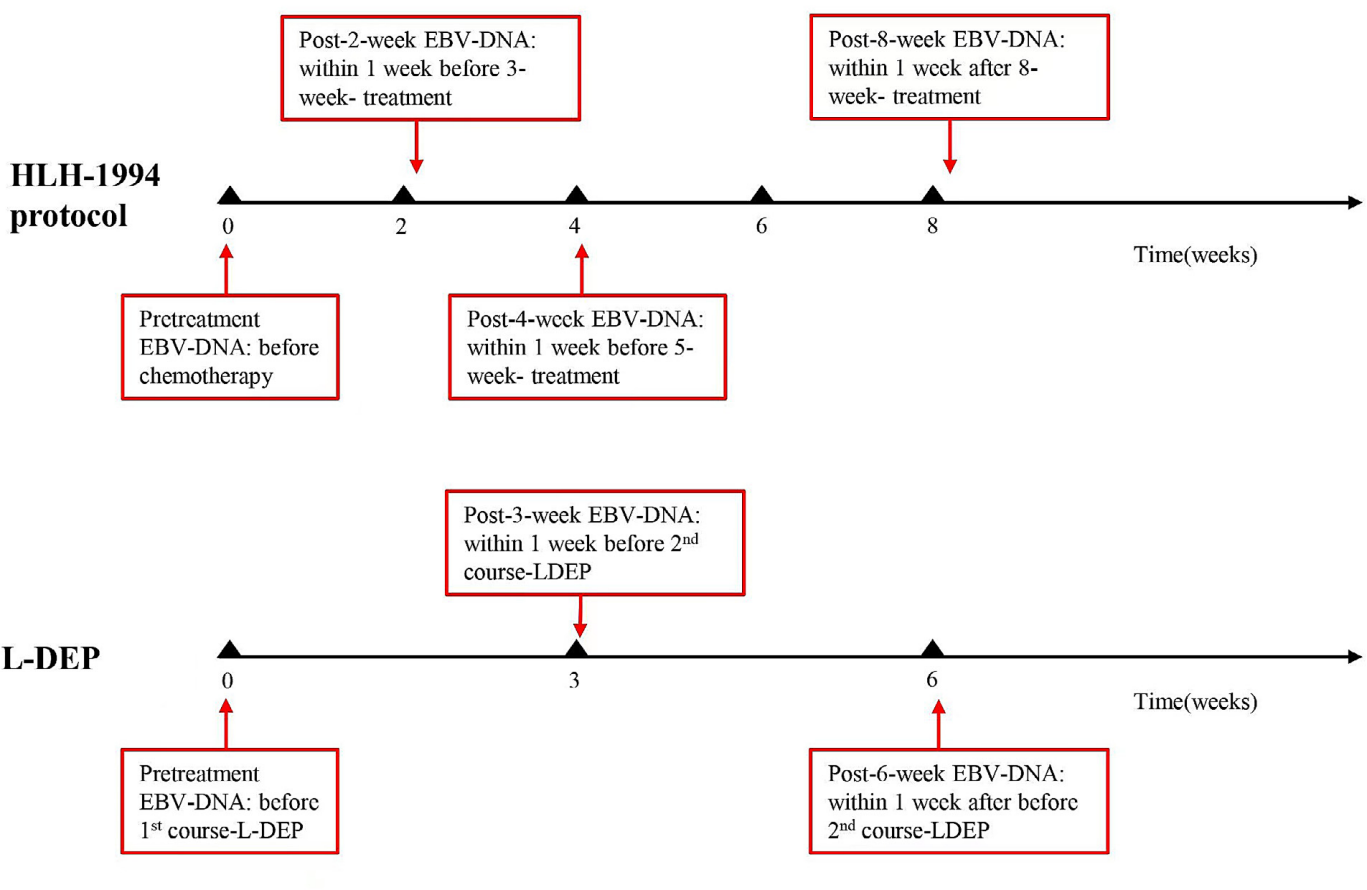
Different time points for monitoring EBV-DNA during treatment.

EBV-DNA was tested at the following time points: at the diagnosis (0 weeks) and 2, 4, and 8 weeks after treatment initiation, as well as when deemed necessary by the clinician (Figure 1). EBV-DNA were tested to identify disease conditions, such as a recurrence of fever, which could not be determined as infections or relapses. Persistent negative means that EBV-DNA tested negative at all of the above time points after treatment. Persistent positive means that EBV-DNA tested positive at all of the above time points. A commercial quantitative EBV PCR assay kit (Sansure Biotech Co. Ltd., Hunan, China) was used to quantify the viral load. The labeled lower detection limit for this assay was 400 copies/ml. EBV-DNA < 400 copies/mL was defined as negative.

### Statistical methods

Statistical analysis was performed using GraphPad Prism 8.0. Rates were compared using Fisher's exact test. *P-*value < 0.05 was considered statistically significant.

## Results

### Patient characteristics and EBV-DNA

A total of 20 cases of EBV-HLH were analyzed, including 16 cases of EBV-infection-associated HLH, 4 cases of EBV-positive lymphoma-associated HLH (two patients diagnosed with T-cell lymphoma, one with extranodal NK/T-cell lymphoma, and one with diffuse large B-cell lymphoma). The median age of onset was 45 months (range, 12–113 months), and there were 7 males and 13 females. All patients received first- and second-line treatment with the modified HLH-94 regimen.

A total of 162 EBV-DNA results were analyzed, including 81 PBMC EBV-DNA results and 81 plasma EBV-DNA results. Pre-treatment plasma EBV-DNA was elevated in all 20 patients. During treatment, the plasma EBV-DNA of seven patients turned negative and remained negative, and none of these patients relapsed. Four patients had persistently positive plasma EBV-DNA, of whom three relapsed during treatment, and one refused to continue treatment after 4 weeks. Nine patients had re-elevated plasma EBV-DNA after turning negative during treatment, and five of these patients had relapses. Table 1 describes the clinical characteristics and outcomes of these 9 patients. Among the 9 patients with EBV-DNA re-elevation after turning negative, the average load of elevated EBV-DNA was 1.11E+04 copies/ml for the 5 patients who experienced recurrence and 1.65E+03 copies/ml for the 4 patients who did not relapse.

**Table 1:** Clinical characteristics and outcomes of the 9 patients with re-elevation of plasma EBV-DNA during treatment.

(*N* = 9)	Malignancy/HLH-related genes	Pretreatment EBV-DNA load (copies/ml)	Pre-treatment		EBV-DNA load of re-elevation (copies/ml)	Recurrence	Post-treatment	Turn to negative	Outcome
P1	-/-	4.08E+04		2 weeks induction	3.64E+03	Yes	L-DEP and HSCT	Yes	Alive
P2	-/PRF1, HBB			8 weeks induction +3 courses of DEP+PD1 inhibitor	3.02E+01	Yes	L-DEP	Yes	Alive
P3	EBV-positive T-cell lymphoma/-	3.30E+02		2 weeks induction +SMILE+CHOP	3.91E+04	Yes	L-DEP and HSCT	Yes	Alive
P4	-/FANCD			2 weeks induction	2.66E+03	Yes	L-DEP	No	Died
P5	CAEBV /RECQL4, LYST	3.47E+03		4 weeks induction	1.03E+04	Yes	HSCT	Yes	Alive
P6	-/-			4 weeks induction +L-DEP	1.87E+03	No	Outpatient follow-up	Yes	Alive
P7	-/-	9.06E+03		4 weeks induction	1.47E+03	No	Continue induction	Yes	Alive
P8	-/-			4 weeks induction	2.66E+03	No	Continue induction	Yes	Alive
P9	-/-	5.48E+04		4 weeks induction	6.04E+02	No	Discharge		Missing

CAEBV, chronic active EBV infections.

### Prognosis

Based on the dynamic changes in plasma EBV-DNA during treatment, 20 patients were divided into three groups: Group A (patients with the sustained positive EBV-DNA during treatment) (*n* = 4); Group B (patients with the EBV-DNA re-elevated after turning negative) (*n* = 9), and Group C (patients with EBV-DNA turning negative and kept negative during treatment) (*n* = 7). Among these groups, 75 % of patients (3/4) in Group A were diagnosed with EBV-positive lymphoma, compared to 11 % (1/9) in Group B and 0 % (0/7) in Group C.

75 % (3/4) of patients in Group A had relapse, with two patients dying after relapse and one surviving after L-DEP chemotherapy, 56 % (5/9) of Group B relapsed, as detailed in Table 1, and none of Group C relapsed, and did not relapse. The relapse rate in Group A was significantly higher than that in Group C (*p* = 0.014), and the relapse rate in Group B was significantly higher than that in Group C (*p* = 0.029).

### Cases

Among the patients included in this study, nine patients experienced a re-elevation of plasma EBV-DNA, despite having turned negative previously following treatment. Out of the nine, five experienced a relapse. The phenomenon of re-elevated plasma EBV-DNA during treatment can indicate either disease relapse or stable disease status. Consequently, individualized treatment strategies become necessary. To illuminate the decision-making process, the authors have presented three cases in which patients experienced a re-elevation of plasma EBV-DNA levels during treatment.

Case 1 was a six-year-old male diagnosed with EBV-HLH. Initially, his plasma EBV-DNA level was 2.01E+04 copies/ml. After two weeks of treatment, the plasma EBV-DNA became negative, but at the four-week evaluation, it increased to 1.47E+03 copies/ml without progression of the disease. As a result, the first-line therapy was continued. Finally, the plasma EBV-DNA became negative at the eight-week evaluation, and CR was achieved after two maintenance treatments (VP-16+ dexamethasone). Chemotherapy was discontinued for four years, and the patient's general condition is good. His plasma EBV-DNA monitor consistently showed negative.

Case 2 was a 4-year-old female diagnosed with EBV-HLH and chronic active EBV infection (CAEBV), with an initial plasma EBV-DNA level of 5.48E+04 copies/ml. The patient's plasma EBV-DNA level turned negative at the 4-week evaluation but increased to 1.03E+04 copies/ml after L-DEP chemotherapy. This was accompanied by a relapse of HLH, evidenced by the presence of fever, cytopenia in two lineages, and elevated levels of serum ferritin and sCD25. Genetic testing revealed two missense variants associated with HLH: c.2131G>A (p.Glu711Lys) in Exon13 of the RECQL4 gene, with a heterozygous variant frequency of 42.78 %, and c.3107C>T (p.Glu1036Ala) in Exon6 of the LYST gene, with a heterozygous variant frequency of 47.72 %. In addition, it was noted that the patient presented with abnormal NK cell function, exhibiting a mere 3 % NK cell activity and decreased NK cell CD107a degranulation prior to treatment. These deficits persisted after treatment, with 3.05 % NK cell activity and continued decreased NK cell CD107a degranulation, suggesting a potential deficiency in NK cell function resembling primary HLH. Subsequently, the patient received allogeneic hematopoietic stem cell transplantation (allo-HSCT) due to disease recurrence. The patient's father was the donor for the transplantation and was also tested for HLH-related genes, revealing a mutation in LYST, which was inherited by Case 2. Furthermore, functional tests, including NK cell activity and CD107a degranulation, were conducted on the father and were found to be normal. After HSCT, the patient experienced cytomegalovirus (CMV) infection and exhibited mild graft-versus-host disease (GVHD) involving the liver, skin, and intestines. Fortunately, she achieved CR after anti-virus therapy and treatment of ruxolitinib and mycophenolate mofetil. The patient has been off treatment for three years and is generally in good health. Regular monitoring of the patient's plasma EBV-DNA levels showed they remained negative.

Case 3 was a two-year-old female diagnosed with EBV-HLH, with an initial plasma EBV-DNA level of 1.85E+05 copies/ml. After four weeks of induction therapy, the plasma EBV-DNA turned negative but subsequently increased to 1.87E+03 copies/ml after L-DEP chemotherapy. Despite the increase in EBV-DNA, the patient did not exhibit any abnormal clinical or laboratory findings. As a result, chemotherapy was discontinued, and the patient has remained in good health without any relapse for over two years, with regular outpatient follow-ups demonstrating consistently negative plasma EBV-DNA levels. The clinical characteristics of the three patients at the time of re-elevation of plasma EBV-DNA are shown in Table 2.

**Table 2:** Clinical characteristics of the 3 patients at the time of re-elevation of plasma EBV-DNA.

Clinical features	Case1	Case2	Case3
Gender	Male	Female	Female
Age(years)	6	4	2
History	No	CAEBV	Recurrent tonsillitis
Fever	No	Yes	No
Lymph nodes/liver/spleen enlargement	No	No	No
WBC(G/L)	5.52	2.15	2.42
ANC(G/L)	2.37	0.66	0.12
HB(g/L)	122	76	121
PLT(G/L)	320	178	157
TG(mmol/L)	2.37	1.3	1.08
LDH(U/L)	207	432	342
SF(μg/L)	126.4	1202.3	174.8
Fib(g/L)	2.97	4.04	0.74
IL-6(pg/ml)	3.25	6.17	14.33
IL-10(pg/ml)	3.86	78.49	6.87
EBV-DNA in PBMCs(copies/ml)	4.77E+04	7.50E+06	7.80E+05
EBV-DNA in plasma (copies/ml)	1.47E+03	1.03E+04	1.87E+03
EBV-sorting	/	B, T, and NK cells were involved	B, T, and NK cells were involved
NK cell activity(%)			11.7
sCD25(U/ml)	9.68		Negative
HLH-related genes	Negative	Missense variant on RECQL4.Exon13 c.2131G>A (p.Glu711Lys) (heterozygous, variant frequency 42.78 %) Missense variant on LYST.Exon6 c.3107A>C (p.Glu1036Ala) (heterozygous, variant frequency 47.72 %)	Negative

CAEBV, chronic active EBV infections; WBC, white blood cell; ANC, absolute neutrophil count; HB, hemoglobin; PLT, platelet; TG, triglyceride; LDH, lactate dehydrogenase; SF, ferritin; Fib, fibrinogen; IL-6, interleukin-6; IL-10, interleukin-10; PBMC, peripheral blood mononuclear cells; NK, natural killer; sCD25, Soluble interleukin-2 receptor alpha chain.

## Discussion

The presentation of EBV-HLH varies widely, from severe infectious mononucleosis that may resolve spontaneously, to progressive diseases requiring immunosuppression, chemotherapy, or even allo-HSCT^1^. It is essential to promptly and accurately evaluate the condition of patients with HLH to choose appropriate treatment methods.

Patients with EBV-HLH often have high plasma EBV-DNA load at diagnosis, and EBV reactivation during treatment often indicates relapse. Re-elevation of plasma EBV-DNA during treatment is common in patients, 45 % (9/20) of patients had re-elevation EBV-DNA after turning negative during treatment in this study. However, the next plan for patients who have re-elevated plasma EBV-DNA has not been clearly proposed in previous studies. Generally, the next treatment strategy has two directions: one is aggressive therapeutic interventions, such as second-line chemotherapy(L-DEP) or HSCT; the other is observing cautiously and monitoring EBV-DNA regularly, to clear EBV relying on the original treatment and immunity.

Based on the present study, further treatment strategy selection can be considered from the following three aspects. Firstly, the level of EBV-DNA elevation is crucial.^11^^,^^12^ In this study, nine patients had re-elevation of EBV-DNA during treatment. The average elevated EBV-DNA load was 1.11E + 04 copies/mL in the five patients with recurrence and 1.65E + 03 copies/mL in the four patients without recurrence. Elevated plasma EBV-DNA levels in patients with relapses were higher than in those without relapses. Zhou et al. found that plasma or PBMCs EBV-DNA load levels could distinguish active and inactive phases of CAEBV in adults.^12^ In EBV-NK-LPDs, patients with plasma EBV-DNA over 4.16E+03 copies/ml were more likely to combine with HLH^12^. In adult EBV-HLH, high levels of EBV-DNA load were significantly related to worse therapy response and poor outcomes.^13^ The recurrence rate was significantly higher in pediatric patients with a plasma EBV-DNA load > 10^3^ copies/mL after two weeks of treatment.^5^ As a result, EBV-HLH patients with excessive EBV-DNA elevation during treatment are more likely to relapse and should prompt an approach with aggressive therapeutic interventions.

Secondly, aggressive therapeutic interventions should also be considered in patients with excessive inflammation and worse prognosis factors. Studies have demonstrated that patients with EBV infection involving T cell or NK cell infection had a worse prognosis compared to those with B-cell-type infection.^14^^,^^15^ In this study, 50 % (6/12) of the patients in this study had T and NK cell involvement; however, no significant prognostic difference was found, likely due to the small number of cases. Cytokines such as sCD25, IL-6, IL-10, TNF-α, and IFN-γ have been identified as reliable indicators for diagnosis of EBV-HLH and evaluation of therapeutic response.^16^ In Case 2, who had relapsed, the re-elevation of EBV-DNA was accompanied by significant increases in IL-6 and IL-10, indicating a state of hyperinflammation. A retrospective study found that TG and total bilirubin levels were risk factors affecting the response rate and prognosis of EBV-HLH.^17^ Additionally, hyperbilirubinemia and hyperferritinemia at diagnosis were also found significantly associated with poor prognosis.^18^ Therefore, when EBV-DNA re-elevation is accompanied by excessive inflammation, timely control is crucial.

Thirdly, in cases where patients are unable to eliminate EBV due to underlying immunodeficiencies, such as primary HLH or malignancies, more intensive and aggressive treatment may be warranted. This could involve escalated chemotherapy regimens, immunomodulatory therapies, or HSCT to address the underlying immune dysfunction and control EBV infection. Impaired lymphocytotoxicity is present in primary HLH and immunocompromised in malignancy-associated HLH^2^. Case 2, who relapsed at the time of EBV-DNA elevation, had two HLH-related genetic alterations, low NK-cell activity (0.17 %) before treatment, and a lower degranulation level of Cytotoxic T lymphocytes (CTL) during activation, which indicated immunodeficiency. In the present study, anti-EBV-CTL activity testing was performed in three of the nine patients with EBV-DNA re-elevation. The two patients with negative anti-EBV-CTL activity both relapsed, while the patients with positive activity did not relapse and subsequently had negative EBV-DNA without therapy. Therefore, when EBV-DNA is elevated again, immune function tests such as anti-EBV-CTL activity may be helpful in evaluation.

EBV not only causes HLH directly but also acts as a trigger for other types of HLH, including the most common primary HLH and lymphoma-associated HLH.^19^ Some primary HLHs, such as XLP-associated HLH, are largely driven by EBV.^2^^,^^20^ EBV infection occurs in over 90 % of patients with NK/T cell lymphoma. A study that included 567 patients with HLH found that the 5-year overall survival rate exceeded 80 % for patients with EBV or other infection-associated HLH, was intermediate for those with FHL or B-cell lymphoma-associated HLH, and poor for those with T/NK cell lymphoma-associated HLH (< 15 %).^19^ Patients with persistent positive plasma EBV-DNA should be further screened for the possibility of underlying lymphoma and primary HLH. Three out of five patients with persistent positive plasma EBV-DNA in this study had a confirmed diagnosis of EBV-positive lymphoma. EBV+T/NK cell lymphoma must be considered as an underlying diagnosis when EBV-HLH does not respond to targeted HLH therapy.^12^ Impaired lymphocytotoxicity is present in primary HLH and immunocompromised in malignancy-associated HLH. If a patient has HLH-associated genetic alterations or is diagnosed with EBV-associated lymphoma, HSCT for immune reconstitution is more beneficial to the patient's prognosis.

In this study, plasma EBV-DNA of cases 1 and 3 were re-elevated without the genetic background of primary HLH or an excessive inflammatory response. In case 1, after 2 weeks of continued maintenance chemotherapy, EBV-DNA turned negative. In case 3, EBV-DNA turned and remained negative under long-term follow-up without maintenance chemotherapy. In case 2, the EBV-DNA was elevated again along with excessive inflammation and immune deficiency to eliminate EBV. Thus, allo-HSCT was selected decisively to save a life in case 2. Therefore, the re-elevation of EBV-DNA cannot simply be recognized as a sign of EBV-HLH recurrence. Inappropriate aggressive chemotherapy can lead to immunodeficiency and compromise the patient's ability to eliminate EBV. At the time of EBV-DNA re-elevation, patients may have recurrence or remain in stable condition. The two states require different treatment strategies. The determination of the disease status and the choice of treatment strategy requires a combination of the degree of elevated EBV-DNA, the intensity of hyperinflammation, and the patient's immune function.

The present study had several limitations. The incidence of EBV-HLH is low and the number of patients is limited in the present work. The authors proposed a concept to highlight the importance of plasma EBV-DNA in disease assessment and treatment selection. Further research is needed to better understand the role of plasma EBV-DNA monitoring in the management of EBV-HLH.

## Conflicts of interest

The authors declare no conflicts of interest.

## Data Availability

Data will be made available on request.
